# Study of the Impact of Radioactivity Detection on the Water Distribution Network Versus the Installation of an Early Warning Network

**DOI:** 10.3390/s26123859

**Published:** 2026-06-17

**Authors:** Natalia Alegría, Igor Peñalva, Charles Pinto, Adriana Merello

**Affiliations:** 1 Energy Engineering Department, Bilbao School of Engineering, University of the Basque Country, 48013 Bilbao, Spain; igor.penalva@ehu.eus (I.P.); amerello001@ikasle.ehu.eus (A.M.); 2 Department of Mechanical Engineering, Bilbao School of Engineering, University of the Basque Country, 48013 Bilbao, Spain; charles.pinto@ehu.eus

**Keywords:** radioactivity, regulations, continuous measurement, equipment, cost of supply

## Abstract

This study investigates the radiological characteristics of drinking water sources managed by the Bilbao Bizkaia Water Consortium (CABB). To this end, the radiological monitoring parameters established by current regulations, as well as those applied by other international organizations, are reviewed and analyzed. In addition, commercially available continuous monitoring equipment is assessed in terms of its suitability for drinking water applications. To identify optimal deployment locations, a comprehensive evaluation of CABB water infrastructure is conducted, with the aim of ensuring radiological safety across the Bizkaia region. Furthermore, an economic assessment is carried out to estimate the potential cost of water supply under abnormal contamination scenarios.

## 1. Introduction

Today, water is an increasingly important resource due to the challenges we face, such as climate change, population growth, potential contamination, etc. Water has always been an essential resource necessary for life and plays a fundamental role in all areas of society [1].

Radiological contamination of drinking water represents a low-probability but potentially high-impact threat to public health, environmental protection, and the continuity of essential services. Although routine radiological monitoring programmes based on periodic sampling are established in most developed countries, these approaches may not provide sufficient responsiveness in the event of accidental releases, malicious contamination, or rapidly evolving radiological incidents. Consequently, several countries and regional authorities have progressively incorporated continuous monitoring systems that are capable of providing real-time information and early warning capabilities. Several national and regional authorities have also progressively incorporated continuous monitoring systems that are capable of providing real-time information and early warning capabilities [2,3]. These systems have been implemented in response to increasing concerns regarding accidental releases, industrial incidents, and potential malicious contamination events affecting water resources.

Examples include the Environmental Radiological Monitoring Network of Catalonia (XVRAC) [4,5], the Environmental Radioactivity Laboratory of Extremadura (LARUEX) [6,7], the TELERAD river monitoring stations operated in Belgium [8,9,10], and various monitoring programmes coordinated by the International Atomic Energy Agency (IAEA) [11,12].

Continuous monitoring networks provide several advantages over conventional laboratory-based surveillance, including reduced detection times, improved situational awareness, automated alarm generation, and enhanced support for emergency response activities. While laboratory analyses remain indispensable for regulatory compliance and radionuclide-specific characterization, real-time sensor networks constitute an additional layer of protection that can significantly improve preparedness against radiological emergencies.

The present work should therefore be understood as a feasibility and deployment study that is focused on the implementation of a radiological early-warning network within the Bilbao Bizkaia Water Consortium (CABB). Rather than proposing a novel detector technology, this study evaluates available monitoring solutions, identifies optimal deployment locations, analyses operational requirements, and assesses the potential economic benefits associated with the early detection of contamination events.

In the event of radioactive contamination of drinking water, this would have a significant impact with immediate consequences for the population; however, in the long term, this becomes more serious, posing considerable risks to human health.

In Bizkaia, the Bilbao Bizkaia Water Consortium (hereinafter CABB) [13] manages water in 98 municipalities, which represent more than 95% of the total population in Bizkaia, carrying out both supply and sanitation tasks.

**Figure 1:**
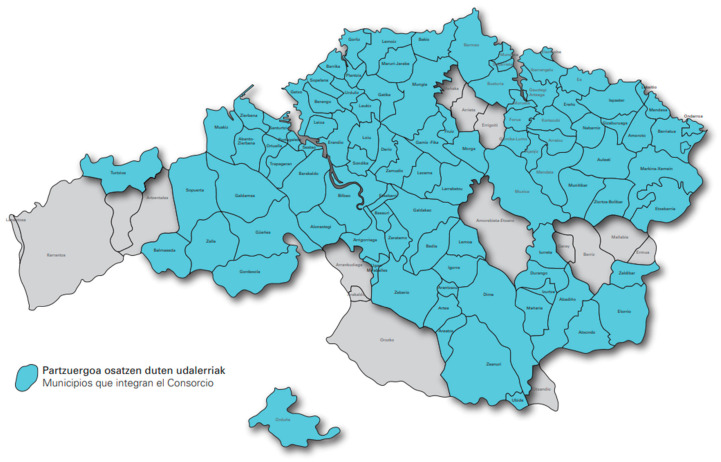
Location of Bilbao [14].

The CABB is a public entity responsible for managing the entire water cycle in the Bilbao region of Bizkaia, Spain. The entity was created on 17 March 1967 to ensure the installation and management of drinking water supply and wastewater sanitation services in the primary network [14,15]. Today, the CABB supplies drinking water to a population of more than 1 million people, as shown in Figure 1. In addition, the Biscay Provincial Council and the Basque Government are members of the CABB Board of Directors, without prejudice to maintaining collaboration agreements with various local entities.

In the case of water collection, the water supplied comes from eight reservoirs: Urrunaga, Ullíbarri-Ganboa, Undurraga, Ordunte, Artiba, Oiola, Nocedal, and Zollo. It is obvious that the importance of water quality in these reservoirs lies in its direct impact on public health.

The CABB Water Hall [16] is a Company Hall that links the CABB with the Bilbao School of Engineering [17], which is part of the University of the Basque Country (EHU) [18]. Within this Hall, a study has been conducted on the impact that radioactive contamination would have on the water supply for the population of Bizkaia. As such, the work has two parts:

- Technical and economic studies of the implementation of an automatic radiological monitoring network for drinking water alerts in Bizkaia.
- Studies on the impact of hypothetical water contamination in Bizkaia and the cost of alternative water supply sources.

Given the importance of comprehensive real-time monitoring of radioactivity in water, the necessary parameters have been studied, as well as the possible methods and technologies implemented in the rest of the world. Likewise, possible locations for the installation of this equipment in the province of Bizkaia have also been studied. Finally, the possible economic cost of these investments has been analyzed.

## 2. Materials and Methods

### 2.1. Existing Laboratories and Measurements Carried out in Them

In Spain, a Royal Decree 3/2023 [19] was published stating that radionuclides must be measured and monitored on an ad hoc basis by taking samples from water intakes, reservoirs, and Drinking Water Treatment Plants (DWTP). Taking into account that sampling and measurements are usually carried out on a monthly basis, and given the concerns about possible radioactive contamination due to current armed conflicts, some regions have already implemented automatic early-warning networks for dammed water.

Some autonomous communities in Spain [20] have had nuclear competences transferred from the central government to corresponding regional governments, such as Extremadura [7], Catalonia [6], Valencia [21], and the Autonomous Community of the Basque Country [22]. Specifically, two of these regions have implemented continuous water monitoring networks:

- In Catalonia, the Environmental Radiological Monitoring Network of the Government of Catalonia (XVRAC) [6] measures and records, in real time, both radiation levels and concentrations of specific radioelements in the air (atmosphere). Additionally, at two of its measuring stations, these measurements are also carried out using the water of the Ebro River.
- In Extremadura, the Environmental Radioactivity Laboratory at the University of Extremadura [7] has 13 continuous sampling stations, 3 of which monitor the status of water.

As Bizkaia is one of the three provinces of the Autonomous Community of the Basque Country [23] and URA is the Water Agency of the Basque Country [24], it could delegate to the community’s water managers. In the case of Bizkaia, management relies almost entirely on the CABB. Therefore, the CABB should study the possible implementation of a water monitoring network in this province.

Worldwide, this concern is also evident. As a result, some laboratories that make up the ALMERA network (Analytical Laboratories for the Measurement of Environmental Radioactivity) [25] for better water control have now implemented specialized equipment for measuring radioactivity in water. These laboratories play a crucial role in monitoring and assessing the radiological safety of drinking water and water resources.

The ALMERA network is a global network of analytical laboratories that measure environmental radioactivity. It was created by the International Atomic Energy Agency (IAEA) [26] with the aim of strengthening the capacity of member countries to monitor and measure radioactivity in the environment, as well as to deal with radiological emergencies. The network consists of 177 laboratories organized into five groups according to their geographical area: Africa, Asia and the Pacific, Europe, the Middle East, and North America and Latin America. All of them are committed to following international standards and procedures to ensure the accuracy and reliability of the data collected on environmental radiation.

The laboratories that have continuous measurement equipment installed are as follows:

- Belgium: The Belgian National Institute for Radioelements (IRE) [27] laboratory, Laboratoire de Mesure de la Radioactivité (LMR), has 12 TELERAD (IMW) river stations for automatic monitoring of river water radioactivity; and the SCK·CEN, Centre d’étude de I’Energie Nucleaire, has more than 250 stations that measure radioactivity in both air and river water 24 h a day throughout the year.
- Greece [28]: The Environmental Radioactivity Laboratory, Hellenic Centre for Marine Research (HCMR), Institute of Oceanography laboratory has two autonomous measuring devices, an autonomous underwater gamma spectrometer (KATERINA II) for marine applications, and GeoMAREA, which is capable of providing continuous, error-free functionality at depths of up to 400 m underwater. It is connected to watertight cabling for real-time data transmission.
- Italy [29]: The ARPA Lombardy, Regional Agency for Environmental Protection of Lombardy, Regional Radiation Protection Center (Agenzia Regionale per la Protezione dell’Ambiente della Lombardia, Centro Regionale Radioprotezione) has fixed monitoring points that operate continuously. Throughout the month, 200 L of water is collected in a special ion exchange resin that comprises properties that retain present radioactive species for analysis by gamma spectrometry.
- Norway [30]: The Norwegian Radiation and Nuclear Safety Authority measures seawater, rainwater, and river water. Most of these measurements are taken as part of the real-time monitoring programmes established on site.

It can therefore be concluded that the implementation of continuous monitoring equipment for rapid alert systems for the population is a reality in many countries.

### 2.2. Inventory of Radionuclides to Be Measured and Available Equipment

In Spain, the RD3/2023 Royal Decree [19] specifies the radionuclides that must be measured, as well as the maximum permitted values for them. Table 1 shows the inventory of these radionuclides and their concentrations.

The Recommended Effective Dose (RED) for one year of intake is due to all radionuclides whose presence has been detected in drinking water, whether of natural or artificial origin, excluding tritium (^3^H), potassium 40 (^40^K), radon 222 (^222^Rn), and the decay products of short-lived ^222^Rn. Summative parameters of all the following radionuclides include ^241^Am, ^14^C, ^60^Co, ^134^Cs, ^137^Cs, ^131^I, ^210^Pb, ^210^Po, ^239^Pu, ^240^Pu, ^226^Ra, ^228^Ra, ^90^Sr, ^234^U, and ^238^U.

The RED is calculated by evaluating the concentrations of the radionuclides measured and the dose coefficients listed in Table A of Annex III of Royal Decree 783/2001 of 6 July [31]. This calculation is made considering an annual intake of 730 L of water for adults.

**Figure 2:**
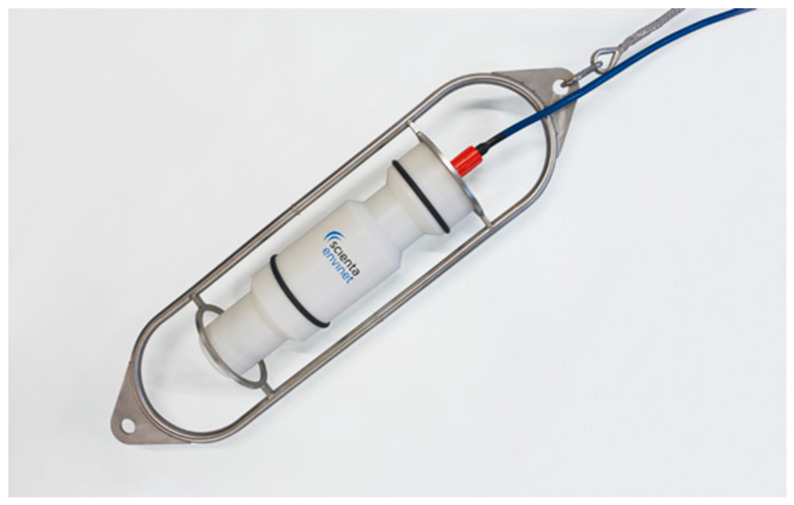
TUNA device with fixed station [33].

Based on the need for continuous measurement in water of alpha activity, beta activity, tritium, and radon, the RED of isotopes established by Royal Decree 3/2023, and additionally, gamma spectrometry, contact was made with several companies working in the field of radioactivity, including Bertin [32], Envinet [33], Berthold [34], Westinghouse [35], Dr. Westmaier [36], Tecnasa [37], Dilus [38], and Saphymo [39], requesting information on possible devices. Only 2 replies were received as follows:

- Envinet [33]: The TUNA device (Scienta Envinet/ENVINET GmbH, Munich, Germany) [33] is a continuous measurement device that can only measure gamma spectrometry. Online monitoring is only possible up to a depth of 50 m. It can be used in fixed stations connected to the electrical grid and LAN, or as a stand-alone solution with solar power and LTE (Figure 2), or with a mobile base for short-term measurements equipped with GPS, LTE, and a battery.

Depending on the desired resolution, the detector glass must be made of one material or another. Table 2 shows the resolution obtained depending on the material.

- Tecnasa [37] proposes a combination of a solid scintillation detector (TECNASA, Tecnologías Asociadas, Madrid, Spain) (from the different commercial brands it represents in Spain, offering NaI, LaBr_3_, or CeBr_3_ detectors) + OSPREY electronics + GENIE2K software, all housed in a waterproof vessel [40] to perform gamma spectrometry, representing a high-performance combination. OSPREY electronics are a fully integrated, high-performance multichannel analyzer (MCA) tube base that contains everything needed to support scintillation spectrometry [41]. GENIE software enables interaction with measurement data, enabling faster decision-making.

Only two of the eight contacted suppliers provided detailed technical and economic information suitable for comparative evaluation. This limitation introduces a potential selection bias and reduces the representativeness of the technology assessment. Nevertheless, both suppliers proposed solutions based on well-established gamma spectrometry technologies that are currently employed in environmental monitoring applications. Therefore, the collected information was considered sufficient for a preliminary feasibility study, although future investigations should include a broader market survey and independent benchmarking of commercially available systems.

The proposed monitoring strategy is based primarily on continuous gamma spectrometry systems because these technologies currently represent the more mature and commercially available solutions for real-time radiological surveillance of water infrastructures. However, gamma monitoring alone cannot fully satisfy all requirements established by Royal Decree 3/2023 [19]. Determinations of gross alpha activity, gross beta activity, tritium concentration, radon concentration, and radionuclide-specific indicative dose assessments must continue to be performed through accredited laboratory procedures. Therefore, the proposed sensor network should be considered an early-warning system designed to complement, rather than replace, the existing regulatory monitoring framework. In practice, continuous gamma monitoring would provide rapid detection of abnormal radiological events, while laboratory analyses would ensure full compliance with regulatory requirements.

#### Limitations of the Selected Monitoring Technologies

The monitoring technologies identified during the market survey are primarily based on gamma-ray spectrometry. These systems provide robust and mature solutions for continuous online monitoring due to their relatively high sensitivity, operational reliability, and capability to identify artificial radionuclides through spectral analysis. However, gamma spectrometry alone cannot fully satisfy all radiological monitoring requirements established by Royal Decree 3/2023 [19].

Regulatory compliance additionally requires the determination of gross alpha activity, gross beta activity, tritium concentration, radon concentration, and radionuclide-specific dose assessments. These parameters generally require laboratory-based analytical techniques involving sample collection, preparation, and dedicated measurement procedures. Consequently, the proposed sensor network should be considered as an early-warning system designed to complement, rather than replace, the existing regulatory monitoring framework.

Table 3 shows the characteristics of the early-warning networks.

### 2.3. Locations to Consider

**Figure 3:**
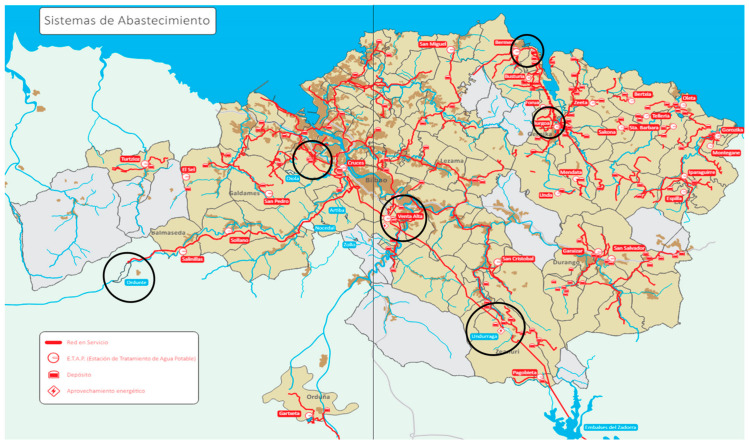
Map of the municipalities belonging to the CABB [43].

In order to select suitable locations for the installation of the measuring equipment, data provided by CABB relating to reservoirs and DWTPs have been checked. Once this information was compiled, the location of each reservoir and DWTP was analyzed, as can be seen in Figure 3. To facilitate visualization of the geographical distribution and to help with the area identification, DWTPs are marked in red and reservoirs in blue. As such, taking into account the maximum capacities and areas covered by each water source, the most relevant ones for the population of Bizkaia will be chosen.

Table 4 provides information about the capacity of the CABB reservoirs, and Table 5 provides information about the CABB DWTPs. Analyzing these data, and according to the location of each facility in Figure 1, installation of a total of 12 devices in the locations identified in Table 6 was decided upon. The main water supply line to the population of Bizkaia comes from the Undurraga reservoir, which is treated at the Venta Alta DWTP, as well as coming from the Ordunte reservoir. Both lines converge and are already in the sanitation phase when arriving at the Galindo Wastewater Treatment Plant (WWTP). By placing devices at the heads and tails of these reservoirs, as well as at the inlets and outlets of each facility, the safety of most of the population would be ensured, leaving only the Busturialdea area free of radioactivity coverage. In order to cover this region, the Burgoa DWTP has been selected, as it is the largest DWTP in the area, and the Lamiaran WWTP was selected as a secondary line in order to maintain continuous control and meet the initial objectives.

**Table 4:** Capacity of the CABB reservoirs [42].

Reservoirs	Capacity [hm^3^]
Ullibarri-Gamboa	146.00
Urrunaga	71.70
Ordunte	22.18
Undurraga	1.79
Oiola	0.82
Artiba	0.63
Zollo	0.32
Nocedal	0.32

**Table 5:** Information related to the CABB DWTPs [43].

DWTP	Location	Municipalities	Population	Supply Capacity [m^3^/d]
Venta Alta	Arrigorriaga	41	838,532	208,128.0
Ordunte—Sollano	Zalla	3	16,578	30,652.7
Garaizar	Durango	7	45,989	12,995.1
Burgoa	Ajangiz	10	22,889	5268.0
Bermeo	Bermeo	1	16,831	5249.0
Basatxu—Cruces	Barakaldo	1	98,783	4752.1
San Cristobal	Igorre	6	7857	2208.7
Oleta	Lekeitio	1	7107	2154.7
Salinillas	Balmaseda	1	7663	1845.4
Gorozika	Ondarroa	1	8277	1833.8
Iparragirre	Markina	5	7239	1805.5
San Salvador	Abadiño	1	7140	1573.7
Busturia	Busturia	3	3881	1261.0
Gartxeta	Orduña	1	4161	943.0
San Miguel	Bakio	1	2816	453.0
Turtzioz	Trucios-Turtzioz	1	531	306.2
Forua	Forua	2	1262	249.2
Montegane	Berriatua	1	1226	237.5
Bertxia	Ispaster	1	741	230.8
Ulla	Ea	1	858	221.0
Urrutxua	Mendata	2	1914	165.0
Zeeta	Ereño	1	287	153.4
Sakona	Nabarniz	1	264	121.4
El Sel	Sopuerta	1	2729	58.3
Telleria	Gizaburuaga	1	204	51.9
Pagobieta	Ubidea	1	182	49.4
Santa Barbara	Amoroto	1	383	28.0
Espilla	Markina	1	5015	7.6
San Pedro	Galdames	1	836	

**Table 6:** Summary of the location proposals for radioactivity control.

Facility Type	Location
Reservoir	Undurraga
	Ordunte
DWTP	Venta Alta
	Burgoa
WWTP	Galindo
	Lamiaran

## 3. Results

### 3.1. Costs of Implementing a Continuous Water Radioactivity Alert System

Based on the data provided by the two suppliers answering our request, the approximate cost of each detection system, electronics, measurement management system, and data transmission system has been estimated at approximately 80,000.00 € per unit (excluding VAT). Considering that twelve units are planned to be installed and that VAT in Spain is 21%, a total initial investment of over 1 M€ is required.

On the other hand, the cost of labour was also studied, since operators are required to work 8 h shifts, covering 24 h a day throughout the year to ensure the continuous operation of the devices and to be on alert. To this end, as shown in Table 7, it was decided to vary the rates according to the day of the week, differentiating between weekdays (249/365), weekend days (104/365), and public holidays (12/365).

Once the type of days and their corresponding quantities and fees were set, the annual cost of the operators was calculated, resulting in a total cost of over 1 M€ per year, specifically 1,235,520.00 €, taking into account that three operators work each day at the six facilities.

Although maintenance costs were not explicitly quantified in this preliminary assessment, a complete lifecycle evaluation should consider detector calibration, preventive maintenance, corrective interventions, spare-part replacement, software updates, telecommunications services, cybersecurity measures, personnel training, and data-management infrastructure. These factors may significantly contribute to the total cost of ownership and should be addressed in future economic studies.

### 3.2. Hypothetical Studies of Water Supply Cost in the Event of Radioactive Contamination

The cost of two possible water supply radioactive contamination scenarios was calculated hypothetically. These studies were carried out to estimate the real cost of catastrophic situations for the population’s water supply and are intended to demonstrate the improvement that would be brought about by the installation of continuous measurement equipment to provide real-time information on water pollution and, as such, prevent situations that are intolerable for the health of the population.

#### 3.2.1. First Scenario: Contamination of Large Reservoirs, the Zadorra System, Accounting for 80% of Greater Bilbao’s Water Supply

In this scenario, water reaching the Venta Alta DWTP from the Zadorra reservoir is contaminated and must be transferred from another facility. The Ordunte reservoir was chosen, which, although located 50 km from the DWTP, has sufficient capacity to meet the demand. Given the geographical location of both facilities, the water must be transported by large tank trucks with a capacity of 45,000 L. Taking into account the amount of contaminated water (208,128 m^3^), a total of 4626 trips must be made to guarantee the supply capacity of the DWTP. The total cost of transporting water per day, taking into account that the cost for the truck is 1.60 €/km, is 370,080.00 €/d. The calculation rationale is as follows:

- Venta Alta DWTP capacity: 208,128 m^3^/d.
- Distance from Ordunte to Venta Alta: 50 km.
- Capacity of the tank truck: 45,000 L.
- Number of trips: 4626.
- Cost of the tank truck: 1.60 €/km.
- Cost of each trip: 80.00 €/trip.
- Total daily cost: 370,080.00 €/d.

#### 3.2.2. Second Scenario: Contamination of All Reservoirs Supplying Water to Greater Bilbao

For this scenario, contamination of the whole water supply system is considered. This would require the daily capacity to be transferred from another area by boat in order to supply Greater Bilbao. Greater Bilbao consumes 283,003.4 m^3^/d; therefore, it was decided to transfer this volume of water by tank ships from the port of Bayonne (to which water from all over Europe would arrive through waterways) to the port of Zierbena over the course of a whole year. Given that tank ships have a maximum capacity of 50,000 m^3^, six trips per day would be required to ensure the supply of the entire population, at a total cost of 240,000 €/d. The calculation rationale is as follows:

- CABB capacity: 283,003.4 m^3^/d.
- Distance between the port of Bayonne and the port of Zierbena: 120 miles.
- Capacity of the tank ship: 50,000 m^3^.
- Number of trips: 6.
- Cost of each trip: 40,000.00 €/trip.
- Total daily cost: 240,000.00 €/d.

## 4. Discussion

The initial investment for a continuous system equipped with six detection systems, each with two devices, amounts to approximately 1 M€. These systems must be monitored and maintained by facility personnel, meaning that annual operating and maintenance costs would amount to 2 M€. Taking into account that its installation is not mandatory, at first glance, it seems like a very high amount simply because of concerns about the impact on the population if it were to happen. However, having to face the consequences of the hypothetical situations presented may give rise to different scenarios, which are outlined below.

### 4.1. Continuous Water Radioactivity Alert System

Implementing a continuous water monitoring system for potential radiological contamination would significantly enhance public safety by providing real-time detection capabilities and enabling rapid response actions. Such a system would allow authorities to identify abnormal radiation levels at their earliest onset, minimizing exposure risks to the population and preventing the distribution of contaminated water through supply networks. By integrating automated alarms, redundant sensors, and secure data transmission protocols, the system would ensure both high reliability and immediate situational awareness for emergency managers. Overall, continuous monitoring strengthens preparedness, supports evidence-based decision-making, and serves as a critical safeguard for protecting human health in scenarios involving radiological hazards.

#### 4.1.1. Sensor Network Architecture and Operational Considerations

The proposed monitoring framework should consider a distributed sensor network composed of multiple radiological monitoring stations deployed at strategically selected locations throughout the water supply infrastructure. Each monitoring node would integrate a gamma-radiation detector, local signal-processing electronics, data-storage capabilities, and communication interfaces.

Measurement data would be transmitted to a centralized supervisory platform through secure communication channels, such as Ethernet, LTE, or equivalent industrial communication technologies. The centralized platform would perform continuous data acquisition, alarm verification, historical data archiving, and real-time visualization for system operators.

To improve operational robustness, future implementations should incorporate redundant communication pathways, automated fault detection, and alarm prioritization mechanisms. In addition, advanced data-fusion strategies could be developed to combine information from multiple monitoring stations, reduce false alarms, and improve event localization capabilities.

Future research should also investigate detector characterization, alarm generation algorithms, anomaly detection techniques, and network performance metrics under realistic operating conditions.

#### 4.1.2. Performance Validation Requirements

The present study focuses on the feasibility of deploying a radiological early-warning network and therefore does not include experimental validation of detector performance. Consequently, parameters such as minimum detectable activity, response time, sensitivity, selectivity, measurement uncertainty, false positive rates, false negative rates, and long-term operational reliability were not quantified.

Future research should include controlled laboratory experiments, field validation campaigns, and simulation-based analyses to evaluate detector performance under representative hydraulic and radiological conditions. Monte Carlo techniques and contamination scenario modeling could be employed to estimate detection probabilities, alarm generation performance, and network robustness under different operational conditions. Such investigations would provide a quantitative basis for optimizing detector selection, sensor placement, and alarm management strategies.

#### 4.1.3. Public Health Risk Reduction

The principal benefit of continuous radiological monitoring lies in the reduced time interval between contamination onset and detection. Earlier identification of abnormal radiological conditions enables authorities to rapidly implement mitigation measures, isolate affected facilities, and prevent the distribution of contaminated water to consumers.

Although a detailed dose-assessment model was beyond the scope of the present feasibility study, a simple illustrative example highlights the potential benefits of earlier detection. Assuming that a contamination event could be detected 24 h earlier through continuous monitoring, the volume of contaminated water distributed to consumers could be substantially reduced. Consequently, population exposure would be proportionally decreased, while emergency response actions could be implemented more rapidly. The actual magnitude of the risk reduction would depend on radionuclide characteristics, contamination levels, hydraulic conditions, water consumption patterns, and operational response times.

Future research should quantify these benefits through radiation dose modeling, probabilistic risk assessment methodologies, radionuclide transport simulations, and health impact metrics. Such analyses would provide a quantitative basis for estimating the reduction in public health risks associated with different monitoring network configurations and alarm response strategies.

### 4.2. Radiological Remediation Strategies After the Fukushima Accident

Following the 2011 accident at Fukushima, Japanese authorities and the operator Tokyo Electric Power Company (TEPCO) implemented a comprehensive water decontamination and management strategy. Groundwater infiltration and reactor cooling operations continuously generated contaminated water; to address this, TEPCO employed filtration systems for 137Cs and 90Sr, followed by a multi-nuclide removal process known as ALPS (Advanced Liquid Processing System), which removes most radionuclides except tritium, producing treated water labeled as “ALPS-treated water” [44,45,46]. After treatment, this water is stored in tanks on site, and starting in August 2023, TEPCO commenced controlled discharges of treated water to the Pacific Ocean, under regulatory oversight [46,47]. Concurrently, large-scale decontamination has included the removal of contaminated soil: roughly 13–14 million cubic meters of earth and ash have been excavated and stored in provisional repositories as part of a broader environmental remediation effort [48,49]. These combined actions—water purification, controlled release, and soil removal—represent a multi-pronged approach to mitigate radiological contamination risks, safeguard water supplies, and prevent further environmental dispersion of radionuclides.

In case of radioactive contamination, all water facilities have to be cleaned using reservoir water, such as from the DWTP and WWTP. An estimate of the length of the water supply and sanitation network of Greater Bilbao could be around 2000 km, which, if contaminated, would need to be cleaned, just as they did in Japan. The cost of decontamination is extremely expensive, and it should be studied in detail in another study.

## 5. Conclusions

This study highlights the importance of having a continuous radiation monitoring system in place for Bizkaia’s water supply infrastructure. Growing concern about possible radioactive contamination scenarios, coupled with the fundamental role that water plays in the public health and well-being of more than one million inhabitants, justifies the need to move towards more efficient, real-time monitoring systems.

Analysis by national and international laboratories shows that the implementation of automatic radiological measurement systems is now an established practice in many countries, demonstrating their reliability and usefulness for the early detection of contamination incidents. Furthermore, the radionuclide inventory required by Spanish regulations reveals that traditional methods based solely on periodic sampling could prove insufficient in the event of sudden incidents, reinforcing the need for continuous monitoring.

The proposed installation of 12 devices, strategically distributed across reservoirs, DWTPs, and WWTPs in the CABB, allows for effective coverage of the main supply lines. This distribution ensures adequate monitoring at critical points and minimizes the risk of contamination going undetected.

From an economic point of view, although the initial investment is around 1 M€ and annual operating costs exceed 2 M€, financial analysis shows that these expenses would be minimal compared to the costs arising from actual pollution scenarios. In the case of partial contamination, as well as in the more extreme case of total contamination, the logistical costs of ensuring water supply would be extraordinarily high, reaching daily figures that make investment in monitoring not only a reasonable alternative, but also a highly profitable one. The NPV, IRR, and payback values obtained reflect a very rapid return on investment and a significant economic benefit in risk scenarios.

Although the present work constitutes a feasibility study rather than a sensor development investigation, it demonstrates the potential value of integrating continuous radiological monitoring technologies into large-scale drinking water infrastructures. The proposed network architecture provides a practical framework for improving preparedness against radiological emergencies while supporting existing regulatory monitoring programmes.

Future research should focus on experimental validation of detector performance, quantitative assessment of public health risk reduction, optimization of alarm generation algorithms, and implementation of advanced data-fusion techniques capable of combining information from multiple monitoring stations in real time.

Overall, the results show that the implementation of a continuous radiation monitoring network not only contributes to protecting the health of the population and the responsible management of an essential resource, but also represents an economically justified decision, especially given the possibility of facing radiological emergencies with significant economic and social consequences.

## Figures and Tables

**Table 1:** Established parametric values [19].

Parameters	Parametric Value	Unit
Total alpha activity	0.1	Bq/L
Total beta activity	1	Bq/L
Radon (^222^Rn)	500	Bq/L
Tritium (^3^H)	100	Bq/L
Recommended Effective Dose (RED) *	0.1	mSv

* RED: Recommended Effective Dose, calculated as the sum of contributions from relevant radionuclides in drinking water, excluding ^3^H, ^40^K, and short-lived radon decay products.

**Table 2:** Comparison of detector technologies for continuous radiological monitoring.

Detector	Energy Resolution (at 662 keV)	MDA	Response Time	Online Monitoring Capability	Advantages	Limitations
NaI (Tl)	7%	Low-moderate	Seconds–minutes	Yes	Low cost, robust, mature technology	Lower spectral resolution
LaBr_3_ (Ce)	3%	Low	Seconds	Yes	Excellent resolution and sensitivity	High cost, intrinsic background
CeBr_3_	4%	Low	Seconds	Yes	Good resolution, low intrinsic background	Higher cost than NaI (Tl)

**Table 3:** Characteristics of the early-warning networks.

Performance Parameter	Importance of Early Warning	Evaluated in This Study	Future Work
Detection limit (MDA)	High	No	Laboratory testing
Response time	High	No	Field validation
Sensitivity	High	No	Laboratory testing
False alarm rate	High	No	Operational trials
Long-term reliability	High	No	Multi-year monitoring
Public health risk reduction	High	No	Dose modeling

**Table 7:** Estimated fees for the operators of the alert system.

	[€/h]	[€/d]
Weekday fee	20.00	160.00
Weekend fee	30.00	240.00
Holiday fee	40.00	320.00

## Data Availability

The original contributions presented in this study are included in the article. Further inquiries can be directed to the corresponding author.

## References

[1] UNESCO (2024). United Nations World Water Development Report. https://www.unesco.org/en/.

[2] Storey M.V., van der Gaag B., Burns B.P. (2011). Advances in on-line drinking water quality monitoring and early warning systems. Water Res..

[3] Frankemölle J.P., Camps J., De Meutter P., Meyers J. (2025). A Bayesian method for predicting background radiation at environmental monitoring stations in local-scale networks. Geosci. Model Dev..

[4] (2024). Xarxa de Vigilància Radiològica Ambiental de Catalunya (XVRAC): Environmental Radiological Surveillance Network.

[5] Generalitat de Catalunya (2023). Competencias de Medio Ambiente. https://govern.cat.

[6] Universidad de Extremadura (2026). Laboratorio de Radiactividad Ambiental (LARUEX).

[7] Junta de Extremadura (2023). Competencias en Seguridad Radiológica. https://www.juntaex.es.

[8] Federal Agency for Nuclear Control (FANC) (2024). TELERAD: Continuous Radiological Surveillance Network in Belgium.

[9] The Belgian TELERAD Early Warning Network for Environmental Radiation Monitoring. https://afcn.fgov.be/fr/system/files/2021-03-15-telerad-en.pdf.

[10] SCK·CEN—Belgian Nuclear Research Centre (2023). Environmental Monitoring. https://www.sckcen.be.

[11] International Atomic Energy Agency (IAEA) (2010). Environmental and Source Monitoring for Purposes of Radiation Protection.

[12] International Atomic Energy Agency (IAEA) (2014). Radiation Protection and Safety of Radiation Sources: International Basic Safety Standards.

[13] Consorcio de Aguas Bilbao Bizkaia (2023). Memoria Anual. https://www.bizkaia.eus/fitxategiak/05/ogasuna/presupuestos/pdf/2023/1699520920827_PRESUP-AURREKONTU%20CABB%202023WEB.pdf.

[14] Consorcio de Aguas Bilbao Bizkaia (2023). Historia y Evolución del Consorcio. https://www.consorciodeaguas.eus/quienes-somos/el-consorcio/.

[15] Consorcio de Aguas Bilbao Bizkaia (2023). Municipios Consorciados. https://aclima.eus/socio/consorcio-de-aguas-de-bilbao-bizkaia/.

[16] (2024). Aula del Agua CABB—Escuela de Ingeniería de Bilbao. Presentación Institucional. https://www.ehu.eus/es/web/bilboko-ingeniaritza-eskola/relaciones_con_la_empresa/aulas_de_empresa.

[17] Universidad del País Vasco (2024). Escuela de Ingeniería de Bilbao. https://www.ehu.eus/es/web/bilboko-ingeniaritza-eskola/.

[18] Universidad del País Vasco (2024). Información Institucional UPV/EHU. https://www.ehu.eus/es.

[19] España (2023). Real Decreto 3/2023. https://www.boe.es/eli/es/rd/2023/01/10/3.

[20] Consejo de Seguridad Nuclear (2023). Competencias Autonómicas. https://www.csn.es.

[21] Generalitat Valenciana (2023). Agencia Valenciana de Seguridad. https://avsre.gva.es/va/.

[22] Gobierno Vasco (2023). Competencias en Protección Radiológica. https://www.euskadi.eus/acreditaciones-proteccion-radiologica/web01-a2inzer/es/.

[23] Gobierno Vasco (2023). Estructura Territorial de la Comunidad Autónoma Vasca. https://www.euskadi.eus.

[24] URA—Agencia Vasca del Agua (2023). Información Institucional. https://www.uragentzia.euskadi.eus.

[25] International Atomic Energy Agency (2023). ALMERA—Analytical Laboratories for the Measurement of Environmental Radioactivity. https://analytical-reference-materials.iaea.org/almera.

[26] International Atomic Energy Agency (2023). Environmental Monitoring Programme. https://www.iaea.org.

[27] Institut National des Radioéléments (2023). Radioactivity Monitoring Systems. https://www.ire.eu/.

[28] Hellenic Centre for Marine Research (2023). Environmental Radioactivity Laboratory. https://www.hcmr.gr.

[29] ARPA Lombardia (2023). Centro Regionale Radioprotezione. https://www.arpalombardia.it/chi-siamo/che-cosa-fa-arpa/radioattivita/.

[30] Norwegian Radiation and Nuclear Safety Authority (2023). Environmental Monitoring. https://dsa.no.

[31] España (2001). Real Decreto 783/2001. https://www.boe.es/buscar/doc.php?id=BOE-A-2001-14555.

[32] Bertin Technologies (2023). Environmental Radiation Monitoring. https://www.bertin-technologies.com/industry/nuclear/environmental-monitoring/.

[33] Envinet GmbH (2023). TUNA Real-Time Gamma Spectrometry System. https://www.envinet.com.

[34] Berthold Technologies (2023). Radiation Measurement Systems. https://www.berthold.com.

[35] Westinghouse Electric Company (2023). Radiation Monitoring Systems. https://www.westinghousenuclear.com.

[36] Dr. Westmeier GmbH (2023). Radiation Detection Equipment. http://www.westmeier-gmbh.de/.

[37] (2023). Tecnasa—Tecnología para el Análisis. Catálogo General. https://www.tecnasa.es.

[38] Dilus Instrumentación (2023). Instrumentación Radiológica. https://dilus.com/es/.

[39] (2023). Mirion Technologies (Saphymo). Environmental Monitoring Systems. https://www.mirion.com.

[40] Mirion Technologies (2023). OSPREY Multichannel Analyzer. https://assets-mirion.mirion.com/prod-20220822/cms4_mirion/files/pdf/spec-sheets/spc-257-osprey-b.pdf.

[41] Jerry Estes Mirion Technologies (2023). Genie 4.0 Spectroscopy Software.

[42] Consorcio de Aguas Bilbao Bizkaia (2023). Embalses—Datos Técnicos. https://www.consorciodeaguas.eus/gestion-del-agua/embalses/.

[43] Consorcio de Aguas Bilbao Bizkaia (2023). ETAP—Datos Técnicos. https://www.consorciodeaguas.eus/gestion-del-agua/instalaciones/.

[44] Anadolu Ajansı (2023). Japan Begins Releasing Treated Water from Fukushima Nuclear Plant into the Ocean.

[45] International Atomic Energy Agency (2023). IAEA Comprehensive Report on the Safety Review of the ALPS-Treated Water at TEPCO’s Fukushima Daiichi Nuclear Power Station.

[46] Nippon.com (2023). What’s Happening with the Release of ALPS-Treated Fukushima Water?.

[47] Nippon.com (2021). Fukushima Decontamination Produces Massive Volumes of Contaminated Soil.

[48] Tokyo Electric Power Company (TEPCO) (2023). Handbook on Treated Water Stored at the Fukushima Daiichi Nuclear Power Station (ALPS-Treated Water).

[49] Tokyo Electric Power Company (TEPCO) (2022). Contaminated Water Treatment and Multi-Nuclide Removal Equipment (ALPS).

